# Pontine and bilateral cerebellar lesion in osmotic demyelination syndrome associated with uncontrolled type II diabetes mellitus: a case report

**DOI:** 10.1097/MS9.0000000000000230

**Published:** 2023-02-17

**Authors:** Suraj Shrestha, Sanjeev Kharel, Sandesh Gautam, Elisha Poddar, Sugat Adhikari, Suman Acharya, Samriddha Raj Pant, Anamika Jha, Rajeev Ojha

**Affiliations:** a Maharajgunj Medical Campus, Tribhuvan University Institute of Medicine, Kathmandu; b Shreegaun Primary Health Care Center, Dang; Departments of c Neurology; d Radiology, Tribhuvan University Institute of Medicine, Kathmandu, Nepal

**Keywords:** extrapontine myelinolysis, hyperglycemia, osmotic demyelination syndrome

## Abstract

**Introduction and importance:**

Osmotic demyelination syndrome (ODS) as a result of the hyperosmolar hyperglycemic state is rare and can present with variable neurological manifestation due to lysis of myelin sheath.

**Case presentation:**

A 44-year diabetic male presented with complaints of sudden onset, progressive bilateral weakness in lower limbs, and slurring of speech for the past 1.5 months. Cerebellar examination showed a bilaterally impaired finger nose test, dysdiadochokinesia, impaired heel shin test, and an impaired tandem gait. MRI brain (T2 and fluid-attenuated inversion recovery sequences) showed high signal intensity in the central pons and bilateral cerebellum. With a diagnosis of ODS with poorly controlled diabetes, he was treated with insulin, metformin, and supportive measures following which his symptoms subsided gradually.

**Clinical discussion:**

A rapid correction of hyponatremia is considered the most common cause of ODS. Variations in plasma glucose levels, a rare cause of ODS, can cause an abrupt osmolality change causing pontine and extrapontine myelinolysis. Prevention of rapid correction of hyponatremia and rapid changes in plasma osmolality in vulnerable patients is the mainstay of treatment.

**Conclusions:**

Clinical features, imaging studies, and monitoring of serum osmolality, serum glucose, and electrolytes aid in diagnosis and favorable outcomes for the patient.

HIGHLIGHTS

- Hyperosmolar hyperglycemic state can rarely cause osmotic demyelination syndrome (ODS).
- Clinical features and relevant MRI findings are diagnostic of the entity.
- Prevention of rapid changes in plasma osmolality and supportive measures are the mainstay of treatment.

## Introduction

ODS has an unusual neurological manifestation due to damage to the myelin sheath of the brain^1^. The classical presentation of this syndrome is central pontine myelinolysis (CPM) involving white matter tracts of the pons, and extrapontine myelinolysis (EPM) involving extrapontine areas^2^. Though the exact pathogenesis remains unclear, rapid correction of hyponatremia might be the potential culprit which is a serious, however, potentially preventable central nervous system demyelinating syndrome^3^,^4^. In addition, hyperglycemic hyperosmolar syndrome along with ketosis can develop into ODS in type II diabetic patients due to infections, noncompliance with treatment, drugs, and other coexisting diseases^5^.

The initial diagnosis of ODS is often difficult due to its variable clinical presentations. Here, we report an unusual case of ODS involving both pons and cerebellum in a chronic alcoholic with hyperglycemia and hypokalemia. The patient was diagnosed with ODS based on his clinical features, supportive laboratory studies, cerebrospinal fluid examination, and brain MRI sequences in the presence of multiple risk factors as potential triggers. This case has been reported in line with SCARE criteria^6^.

## Presentation of case

A 44-year male presented with complaints of sudden onset, progressive bilateral weakness in lower limbs, and slurring of speech for the past 1.5 months. He had unsteadiness, swaying on both sides while walking, and nasal regurgitation while feeding. These symptoms were progressive for about 10 days, then became static. Owing to travel restrictions amidst the COVID-19 pandemic, the patient visited a local health center where he was prescribed some oral medications. Following the episode, he developed abdominal distension, diarrhea, polyphagia, and polydipsia for 1 month. For the past 5 days, the patient had reduced talk, increased somnolence, and occasional irrelevant talk in the evening. There was no headache, neck pain, facial weakness, fever, loss of vision, loss of consciousness, or seizure. His bowel and bladder habits were normal. Moreover, he did not give a history of any form of trauma, surgery, medicine intake, vaccination, and exposure to toxins or heavy metals. There was no family history of similar illness or any neurological disorders.

The patient was diagnosed with type 2 diabetes mellitus, chronic liver disease secondary to alcohol intake with portal hypertension and ascites 1 year back. The patient was a chronic alcoholic (400 g/day for the last 20 years) and an occasional smoker, left for 2 months. He was taking insulin (glargine 34 U once daily) and linagliptin/metformin (2.5/500 mg twice a day) until 15 days before the onset of the symptoms, following which he left using insulin on his own and also missed the physician’s follow-up.

On examination, he was icteric and had bilateral pedal edema. While assessing the neurological status, he was cooperative and following commands, oriented to person but not to time and place. Pupils were round, regular, and reactive along with intact extraocular eye movements and normal cranial nerve examination. He had cerebellar dysarthria with scanning speech. On motor examinations, all limbs had normal tone with a power of 5/5 (Medical Research Council) across all major joints. The plantar response was bilaterally upgoing. The rest of the superficial and deep tendon reflexes were normal. Cerebellar examination showed a bilaterally impaired finger nose test, dysdiadochokinesia, impaired heel shin test, and an impaired tandem gait. There were no flapping tremors. Initially based on the history and clinical presentation our diagnoses included hepatic encephalopathy, ODS, and Wernicke’s encephalopathy.

On laboratory investigations, complete blood count and renal function test were in the normal range. Random blood sugar was 15 mmol/l and glycated hemoglobulin was 15.5%, corrected sodium 140 mEq/l, and potassium 3.0 mEq/l. Prothrombin time/international normalized ratio was 16.9/1.26, serum albumin 3.8 g/dl, total protein: 6.2 g/dl, alanine aminotransferase 80 IU/l, aspartate aminotransferase 152 IU/l, serum ammonia level 30 µmol/l and gamma-glutamyl transferase 323 IU/l, while viral serology markers were negative. Total, direct, and indirect bilirubin levels were normal. Vitamin B_12_ level was 450 pg/ml. Urine analysis showed the presence of glucose without ketone bodies. Lumbar puncture was done and the subsequent cerebrospinal fluid examination showed no abnormalities.

**Figure 1:**
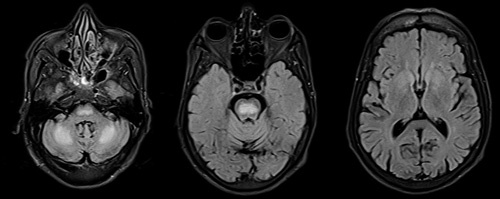
Fluid-attenuated inversion recovery axial images showing hyperintensity in bilateral pons and cerebellum with normal peripheral pontine and basal ganglia region.

**Figure 2:**
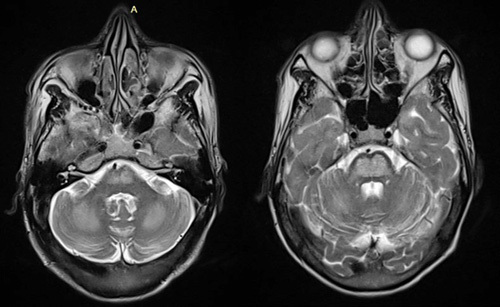
T2 axial images showing hyperintensity in bilateral pons and cerebellum.

Due to an inconclusive diagnosis clinically, an MRI brain was done. T2 and fluid-attenuated inversion recovery brain sequences showed high signal intensity in the central pons and bilateral cerebellum, small vessel disease, and mild cerebral atrophy (Figs. 1 and 2). With these features, a diagnosis of ODS with poorly controlled diabetes was made. During the hospital stay, he was treated with insulin glargine and metformin which gradually controlled his blood glucose level (Table 1). Other medications used were normal saline, methylcobalamin, and thiamine. Gait stabilization physiotherapy was regularly done. After 18 days of hospital stay, the patient was discharged with delirium fully improved, mild dysarthria, and unsteadiness but able to walk without support. On follow-up after 2 months, the patient only had a mild gait problem with no other issues.

**Table 1:** Changes in parameters during the hospital stay

	At emergency	Day 1	Day 2	At the time of discharge	Reference range
Glucose (mmol/l)	–	15	–	8.8	3.8–7.8
Sodium (mEq/l)	130.3	129	133	135	135–146
Potassium (mEq/l)	3.0	3.0	3.2	3.9	3.5–5.2
Creatinine (μmol/l)	–	61	57	71	60–115

## Discussion

ODS is usually depicted in only alcoholics and patients with rapid shifts in osmolarity^7^,^8^. It is a rare condition with unknown incidence and is frequently underdiagnosed. A rapid correction of hyponatremia is considered the most common cause because of the inability of the cells to readapt quickly to higher osmolarity increasing their risk of lysis mainly oligodendrocytes^9^. Similarly, variation in plasma glucose levels can also cause an abrupt osmolality change causing pontine and EPS^10^.

Velver *et al.* 11 showed a rare association between ODS and hyperosmolar hyperglycemic state and its pathophysiological mechanism by rapid hypertonic insult. Our patient’s clinical presentation along with high glycated hemoglobulin and fluctuating serum glucose suggests prolonged hyperglycemia. This suggests fluctuations in glucose concentration adjusted to normal through rapid correction by hydration and insulin therapy have caused myelinolysis of bilateral pons, cerebellum, and medulla. Hyperosmolar hyperglycemic state outweighs the rapid correction of hyponatremia in our case for causation of symptoms as rapid correction of his hyponatremia was not done. This notion is supported by patients’ impaired mental status and ataxia. We suppose ODS might have developed in our patients 1.5 months back, and aggravated by hyperglycemia causing excessive sleepiness with episodes of irrelevant talk.

The risk factors associated with this rare demyelinating disorder are malnutrition, chronic alcoholism, primary adrenal insufficiency, prolonged use of diuretics, hypokalemia, hyperglycemia, fluid resuscitation, hemodialysis, and liver transplant^12^. Our patient had multiple risk factors like chronic alcoholism, hyperglycemia, and malnutrition. However, serum ammonia level and vitamin B_12_ level were within normal range.

The clinical features of CPM and EPM are variable. Classically, CPM has a biphasic course with seizures or encephalopathy seen initially, followed by severe deterioration causing dysarthria, dysphagia, oculomotor dysfunction, and variable degrees of quadriparesis. Rarely, locked-in syndromes have been reported^2^. In contrast, EPM shows various extrapyramidal symptoms and movement disorders^13^. In our case, the pons and cerebellum were involved causing dysarthria, dysphagia, and ataxia.

Though the diagnosis of ODS can be made in the background of rapid hyponatremia correction or a hypertonic insult along with clinical and radiological features, it is important to rule out other differentials including stroke, primary brain tumors, metastases, encephalitis, meningitis, Wernicke encephalopathy, hepatic encephalopathy, and multiple sclerosis^14^. All these differentials were considered and ruled out before a diagnosis of ODS was made in our case.

The current standard diagnostic method of ODS includes clinical features and relevant MRI features. MRI findings may not be seen in early scans and can take about 10–14 days after the onset of symptoms^12^. In our case, MRI was done on our patient 3 days after the admission and 1.5 months after the symptom onset. Pontine demyelination is the hallmark of this syndrome, but isolated extrapontine lesions or combined CPM and EPM have also been reported. Brain MRI typically shows hyperintense lesions in the central pons or associated extrapontine structures on T2-weighted and fluid-attenuated inversion recovery sequences with the corresponding hypointensity on T1-weighted sequences^2^. Extrapontine lesions of ODS often show symmetrical lesions at various sites like basal ganglia, white matter, and cerebellum^15^. In this case, bilateral brain lesions in the pons and cerebellum were suggestive of ODS.

Due to the lack of definitive large studies, there is no proven treatment for ODS. Prevention of rapid correction of hyponatremia and rapid changes in plasma osmolality in vulnerable patients is the mainstay. Various secondary complications of neurological impairment, aspiration pneumonia, ascending urinary tract infections, venous thrombosis, and pulmonary embolism should be minimized and taken care of^16^,^17^. The increased understanding of the pathophysiology of the disease and the use of MRI has aided in early diagnosis improving the outcome of the disease^17^. Though treatment is supportive but additional therapies like intravenous immunoglobulin and plasmapheresis were found to be effective in some case reports^18^. However, the prognosis of ODS is still poor with a high mortality rate. Also, coma and irreversible sequelae may also occur in surviving patients. ODS has a variable outcome and is unpredictable from existing clinical or radiological features^18^,^19^.

## Conclusions

ODS can have variable presentations associated with involved brain areas. Precautions taken during electrolyte balances, abstinence from the risk factors, and physician awareness of the rapid change of serum osmolality and serum glucose are important. Thus, timely diagnosis, supportive care, and prevention of complications can be a cornerstone for a favorable outcome for the patient.

## Ethical approval

Not applicable.

## Consent for publication

Written informed consent was obtained from the patient for publication of this case report and accompanying images. A copy of the written consent is available for review by the Editor-in-Chief of this journal on request.

## Sources of funding

None.

## Authors’ contribution

S.S., S.K., R.O., S.G., S.A., and E.P. were involved in the study concept, data collection, and writing of the manuscript. R.O., S.R.P., and S.A. were involved in the treatment and reviewing of the manuscript. A.J. provided the necessary radiological images and reviewed the manuscript. All the authors were involved in the final review of the manuscript.

## Conflicts of interest disclosure

The authors declare that they have no financial conflict of interest with regard to the content of this report.

## Research registration unique identifying number (UIN)

Not applicable.

## Guarantor

Suraj Shrestha.

## Provenance and peer review

Not commissioned, externally peer-reviewed.
